# Chicken hepatic response to chronic heat stress using integrated transcriptome and metabolome analysis

**DOI:** 10.1371/journal.pone.0181900

**Published:** 2017-07-31

**Authors:** Sara F. Jastrebski, Susan J. Lamont, Carl J. Schmidt

**Affiliations:** 1 Department of Animal and Food Sciences, University of Delaware, Newark, DE, United States of America; 2 Department of Animal Sciences, Iowa State University, Ames, IA, United States of America; Wageningen UR Livestock Research, NETHERLANDS

## Abstract

The liver plays a central role in metabolism and is important in maintaining homeostasis throughout the body. This study integrated transcriptomic and metabolomic data to understand how the liver responds under chronic heat stress. Chickens from a rapidly growing broiler line were heat stressed for 8 hours per day for one week and liver samples were collected at 28 days post hatch. Transcriptome analysis reveals changes in genes responsible for cell cycle regulation, DNA replication, and DNA repair along with immune function. Integrating the metabolome and transcriptome data highlighted multiple pathways affected by heat stress including glucose, amino acid, and lipid metabolism along with glutathione production and beta-oxidation.

## Introduction

Heat stress can affect multiple tissues as an organism alters its physiology to remain viable in the face of this challenge. The physiological adaptations can be manifested at many levels including changes in behavior, and molecular changes at the genomic, transcriptomic, proteomic, and metabolomic level. In response to heat stress, animals will typically seek a cooler environment, along with increasing blood flow to the skin, sweating, panting, and other responses that reduce their body temperature [1]. At the molecular level, a major response to acute heat stress is increased expression of heat shock protein (HSP) genes that encode molecular chaperones responsible for stabilizing protein structures at elevated temperatures [2]. Additional responses to acute heat stress include changes in protein translation and expression of genes affecting the cell cycle, DNA replication and DNA repair [3]. Chronic heat stress can lead to other responses that acclimate the organism to continuous thermal challenge.

The liver is important for maintaining homeostasis of important nutrients, synthesizing bile for solubilizing fats, and providing important circulatory proteins such as albumin and clotting factors. It is reasonable to hypothesize that the liver will be responsive to heat stress due to its central role in maintaining the overall metabolism of the organism. Typically, animals decrease feed consumption under heat stress [1] so a major role of the liver will be to maintain levels of circulating nutrients such as glucose and triglycerides in the face of this challenge. Additionally, hyperthermia triggers oxidative stress [4] suggesting the liver may respond by increasing production of biochemical anti-oxidants [5].

The objective of the present study was to mimic a heat wave by subjecting birds to a cyclic heat stress at 35°C for 8 hours per day. This temperature causes a typical heat stress response including increased panting, wing spreading, lethargy, decreased feed consumption and increased water consumption. This study aimed to identify hepatic transcriptomic and metabolomic responses to chronic heat stress in a rapidly growing broiler chicken line. Changes in the transcriptome indicated responses affecting the cell cycle, DNA replication, DNA repair, and immune functions. Integrated analysis of the metabolome and transcriptome demonstrated responses affecting glucose, lipid, glutathione, amino acid and endocannabinoid levels. Taken together, application of these high-throughput methods provided deep insight into hepatic response to heat stress.

## Results & discussion

### Transcriptome

**Fig 1:**
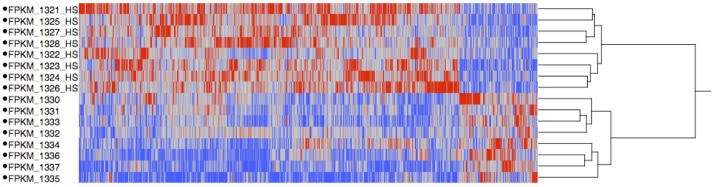
Hierarchical clustering of heat stress and thermoneutral transcriptome libraries from Ross708 liver samples 28 days post-hatch. Under a given condition (heat stress or control) red indicates up-regulation and blue indicates down-regulation. The heat stress and thermoneutral libraries create two separate clusters and the abundance of red in the heat stress libraries likely indicates a robust response to heat stress.

**Fig 2:**
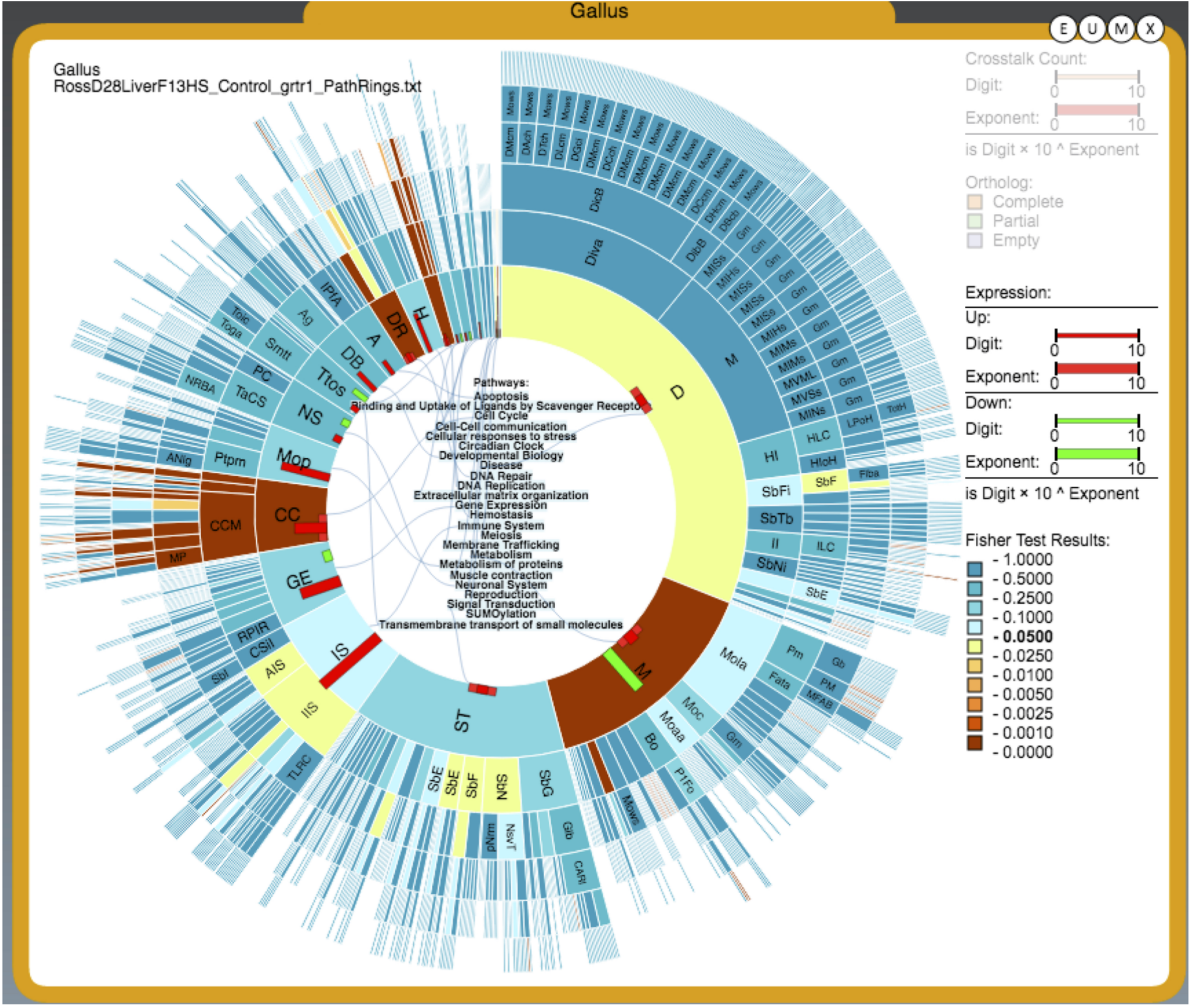
PathRings depiction of liver transcriptome data. Each segment of the inner ring indicates a pathway. Outer rings equate to more specific pathways. Significance is determined by the Fisher’s Exact test and is indicated by color where blue is insignificant and yellow to maroon is significant (0.025–0.001, respectively). Significantly affected pathways were (M) Metabolism, (CC) Cell Cycle, and (DR) DNA Repair.

**Fig 3:**
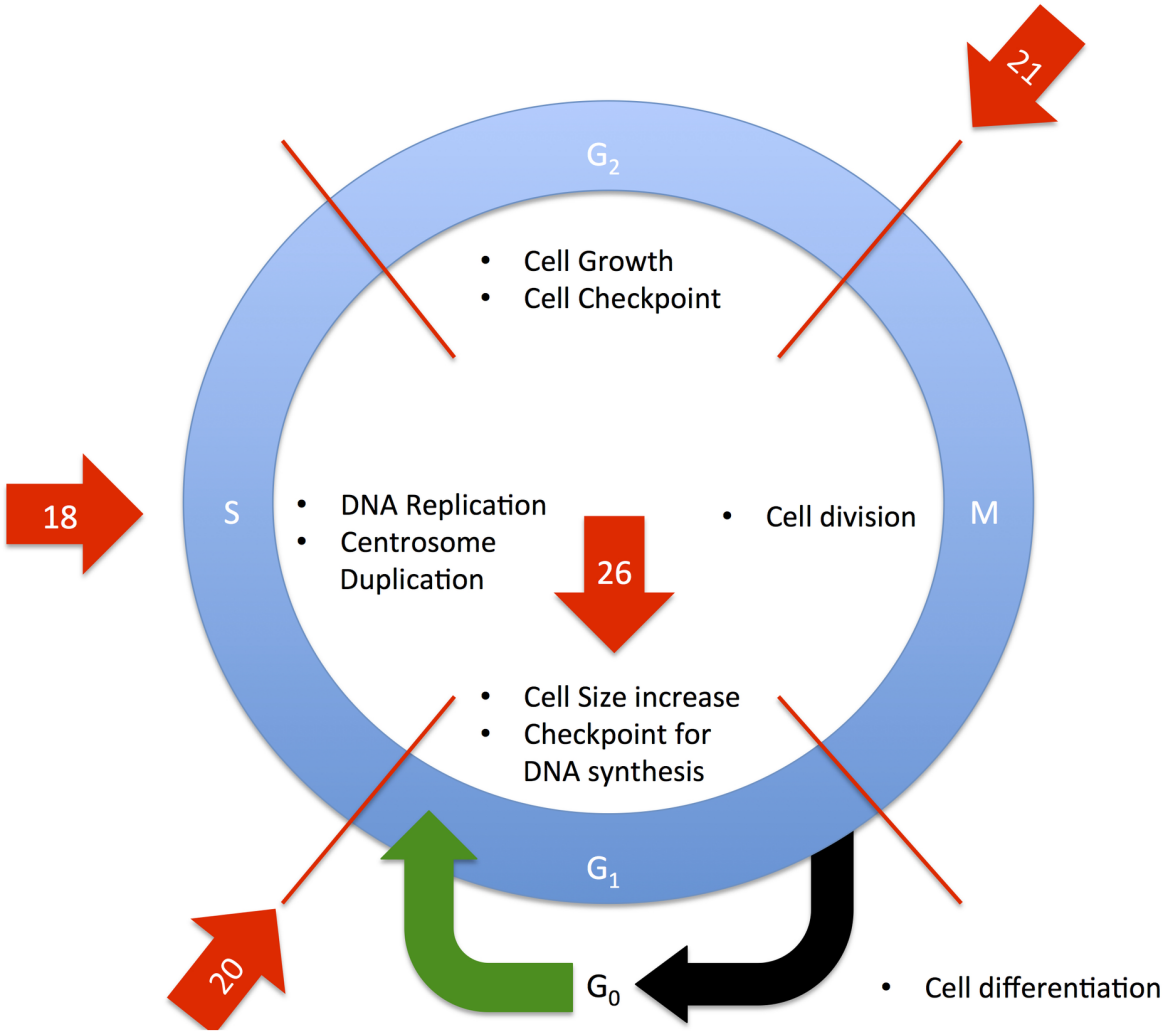
Depiction of the cell cycle. Red arrows indicate parts of the cell cycle affected by heat stress. Red lines indicate transitions between stages of cell cycle. Numbers indicate number of genes at that specific stage of the cell cycle enriched in heat stress as identified by WebGIVI. The stages of cell cycle affected by heat stress include the G1 phase, G1/S phase transition, S phase, and G2/M phase transition.

**Fig 4:**
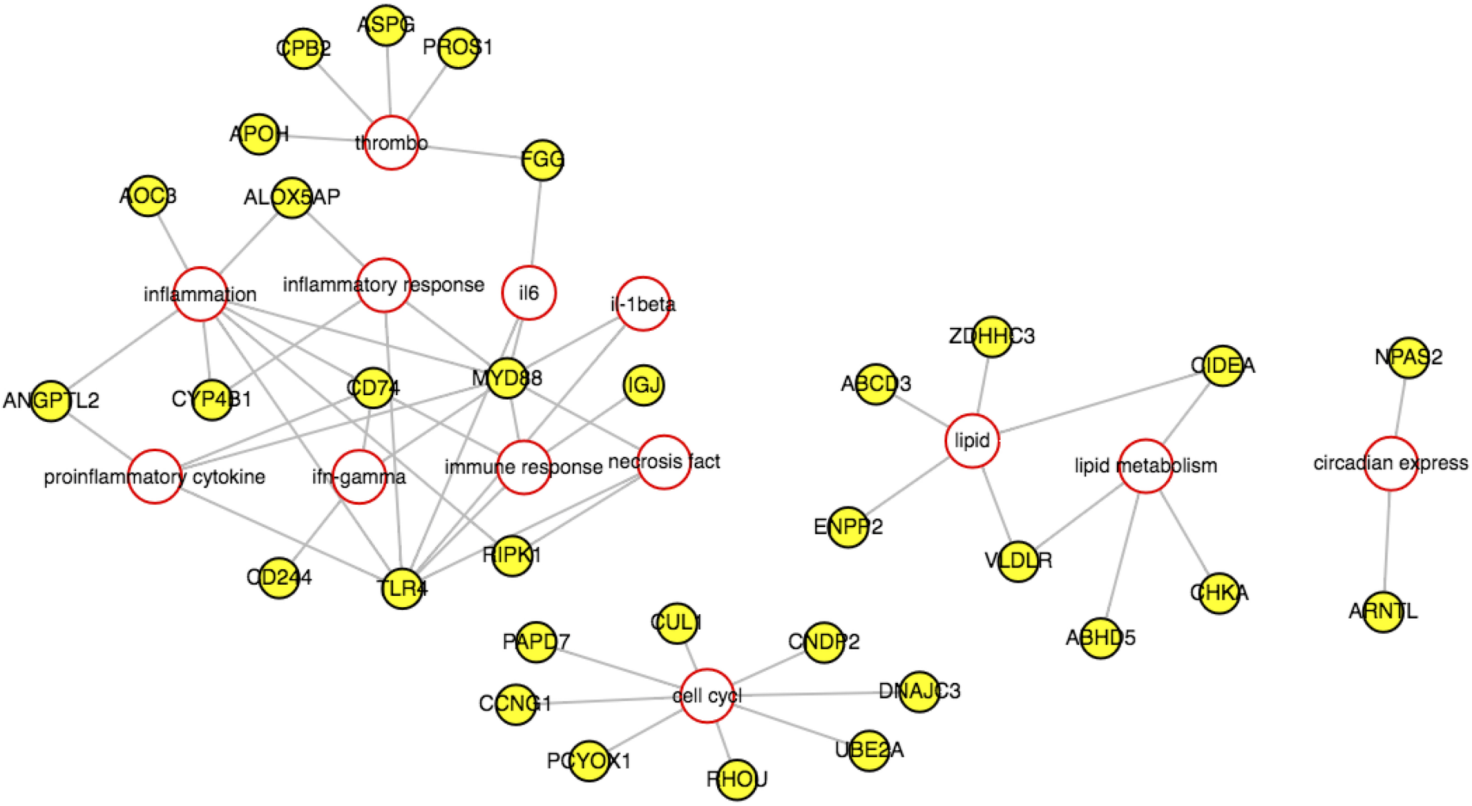
WebGIVI cytoscape output results for thermoneutral transcriptome data from Ross708 liver samples 28 days post-hatch. Yellow circles indicate Genes and red-outlined circles indicate iTerms. Most common iTerms are associated with proinflammatory response, lipid metabolism, cell cycle and circadian rhythm.

A total of 1299 genes were identified as being significantly differentially expressed genes (DEG) with at least one mean FPKM value being greater than one. A total of 1117 genes were enriched in the heat stress condition and 182 enriched in the thermoneutral condition. This suggests that after one week of cyclic heat stress, the liver is producing a robust response to heat stress due to the difference in number of DEG in heat stress and thermoneutral conditions. Hierarchical clustering was used to determine the relationship between the genes’ expression and libraries (Fig 1). Heat stress and thermoneutral libraries create two separate clusters. The top 500 heat stress enriched genes were input into AmiGO2 and the significantly enriched terms are displayed in S1 Table. The top 10 significantly enriched categories were terms associated with cell cycle and DNA repair. Approximately 15% of all the significant terms were associated with cell cycle. PathRings identified cell cycle as a significant pathway, with 37 genes identified as being involved in cell cycle, all enriched in heat stress. PathRings also identified 20 genes involved in metabolism, with 14 enriched in heat stress and 6 enriched in thermoneutral (Fig 2). Fig 3 depicts the cell cycle and identifies parts of the cell cycle that were affected. It appears the major cell checkpoints as well as the G1 and S phases were impacted by heat stress. This is consistent with literature which states that the cell cycle is affected by heat stress [6, 7]. The G1 phase is particularly important for assessing the extracellular environment and determining whether conditions are favorable to divide, and the cell can prolong the G1 phase if conditions are unfavorable [8]. A reasonable hypothesis is that the liver is arresting cell cycle at various stages to wait for favorable conditions to divide. All 182 genes enriched in thermoneutral livers were input into AmiGO2 (S2 Table). It is important to note that enrichment in thermoneutral indicates that the genes are actually down-regulated under heat stress. The most common terms were associated with immune function and lipid catabolism. From WebGIVI, iTerms most commonly associated with down-regulated genes include lipid rand immune function terms (Fig 4). The lipid-associated genes appear to be involved in lipolysis rather than lipogenesis, which is consistent with other reports [9]. Genes that are down-regulated in heat stress affecting the cell cycle appear to be involved in regulating cell proliferation. As a major function of the liver is to maintain homeostasis, it may be dampening its immune function to redirect resources to metabolic functions such as glycogenolysis and lipogenesis. It may also be dampening inflammatory response so as not to increase body temperature.

Seven genes associated with circadian rhythm were identified in the differential gene list in both conditions. Neuronal PAD domain protein 2 (NPAS2) and aryl hydrocarbon receptor nuclear translocator-like (ARNTL) were down-regulated by heat stress. Period circadian clock 2 (PER2), period circadian clock 3 (PER3), prokineticin 2 (PROK2), TEF, PAR bZIP transcription factor (TEF), and basic helix-loop-helix family member e41 (BHLHE41) were enriched in heat stress. ARNTL, NPAS2, PER, and CRY are involved in a complex feedback loop that likely regulates the peripheral liver clock. It is known that acute heat stress resets the circadian clock [10, 11]. Resetting the circadian rhythm could synchronize clock genes to the heat stress cycle, causing the circadian rhythm response to adapt to cyclic heat stress. Clock genes function in many different biological processes including cell cycle and metabolism. The differential expression of these genes between conditions may directly modulate the cell cycle and metabolism changes. More research is needed to determine the exact implication of the effect of heat stress on circadian rhythm.

Deiodinase Iodothyronine Type II (DIO2) was down regulated in heat. DIO2 converts T4 to active T3, which increases the body’s basal metabolic rate and increases oxygen consumption and energy breakdown. Decrease in feed consumption likely contributes to this decrease in DIO2 expression. This is consistent with Coble et al., where they found DIO2 had a two-fold decrease in expression in broiler livers under heat stress [12]. Deiodinase Iodothyronine Type 1 (DIO1) was not significantly different between the two conditions, and is the deiodinase type that is predominantly expressed in the liver.

### Integrated transcriptome and metabolome

#### Glycogenolysis/Gluconeogenesis

**Fig 5:**
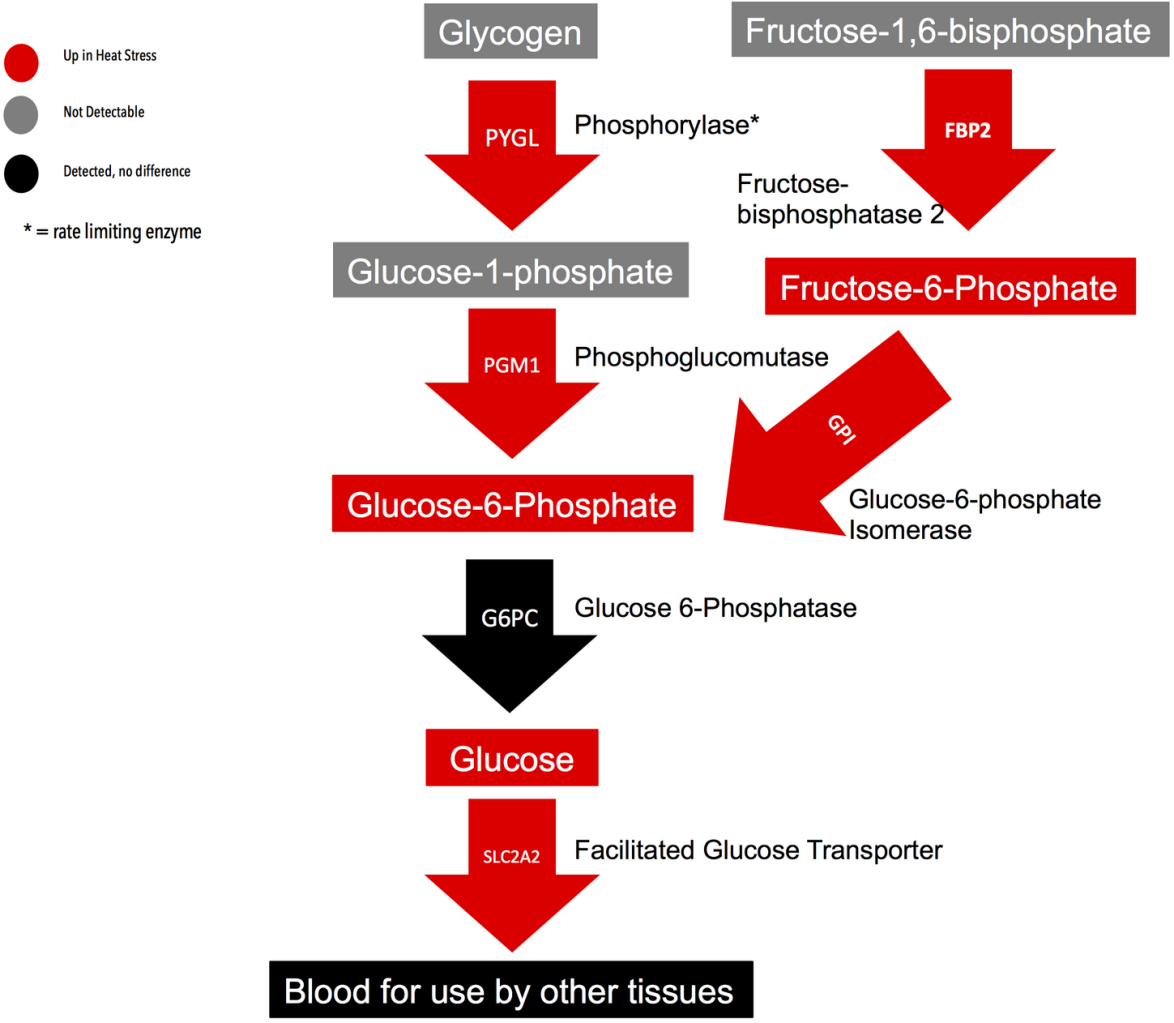
Depiction of glycogenolysis and gluconeogenesis pathways with integrated metabolomic and transcriptomic data from Ross708 liver samples 28 days post-hatch. The rectangles indicate metabolites and the arrows indicate genes. The legend indicates whether a gene or metabolite was detected and if it was up in heat stress or unchanged between conditions.

An important function of the liver is maintaining homeostasis in the face of stress by controlling blood levels of metabolites such as sugars, lipids, and amino acids. During heat stress, chickens reduce feed intake and the liver responds by increasing the production and release of nutrients. Glucose is one essential nutrient as it acts as the major energy source for many tissues, most importantly the brain. The liver can provide glucose systemically either by the breakdown of stored glycogen or through gluconeogenesis. Under heat stress, metabolome and transcriptome data indicate that both glycogenolysis and gluconeogenesis have been elevated in the liver. Fig 5 shows the glycogen metabolism pathway, with integrated transcriptomic and metabolomic data. While glycogen was not detected in the metabolic methodologies, several intermediates cleaving glucose-1-phosphate (G1P) from glycogen were detected. The enzyme that performs these cleavages, phosphorylase, was also elevated in the heat stress samples. Adenylyl Cyclase 9 (ADCY9) was identified from the transcriptome as being enriched in heat stress. This enzyme generates cyclic AMP (cAMP), which activates protein kinase A (PKA). Subsequent phosphorylation by PKA converts phosphorylase from the inactive form to the active form. Metabolites also elevated in livers from heat stressed chickens included glucose-6-phosphate (G6P) and glucose. Phosphoglucomutase (PGM1), which is responsible for transforming G1P to G6P, was elevated by heat stress, while glucose-6-phosphatase (G6PC) was unchanged between conditions. Elevated PGM1 could lead to the observed increased levels of G6P in the heat stressed livers. Increased gluconeogenesis was indicated by the elevated levels of fructose-6-phosphate and the enzyme that transforms it to G6P, fructose-bisphosphatase 2 (FBP2). Finally, we note that transcription of the liver facilitated glucose transporter solute carrier family 2 member 2 (SLC2A2) was elevated during heat stress suggesting an increase in hepatic glucose export.

#### Fatty acid metabolism

**Fig 6:**
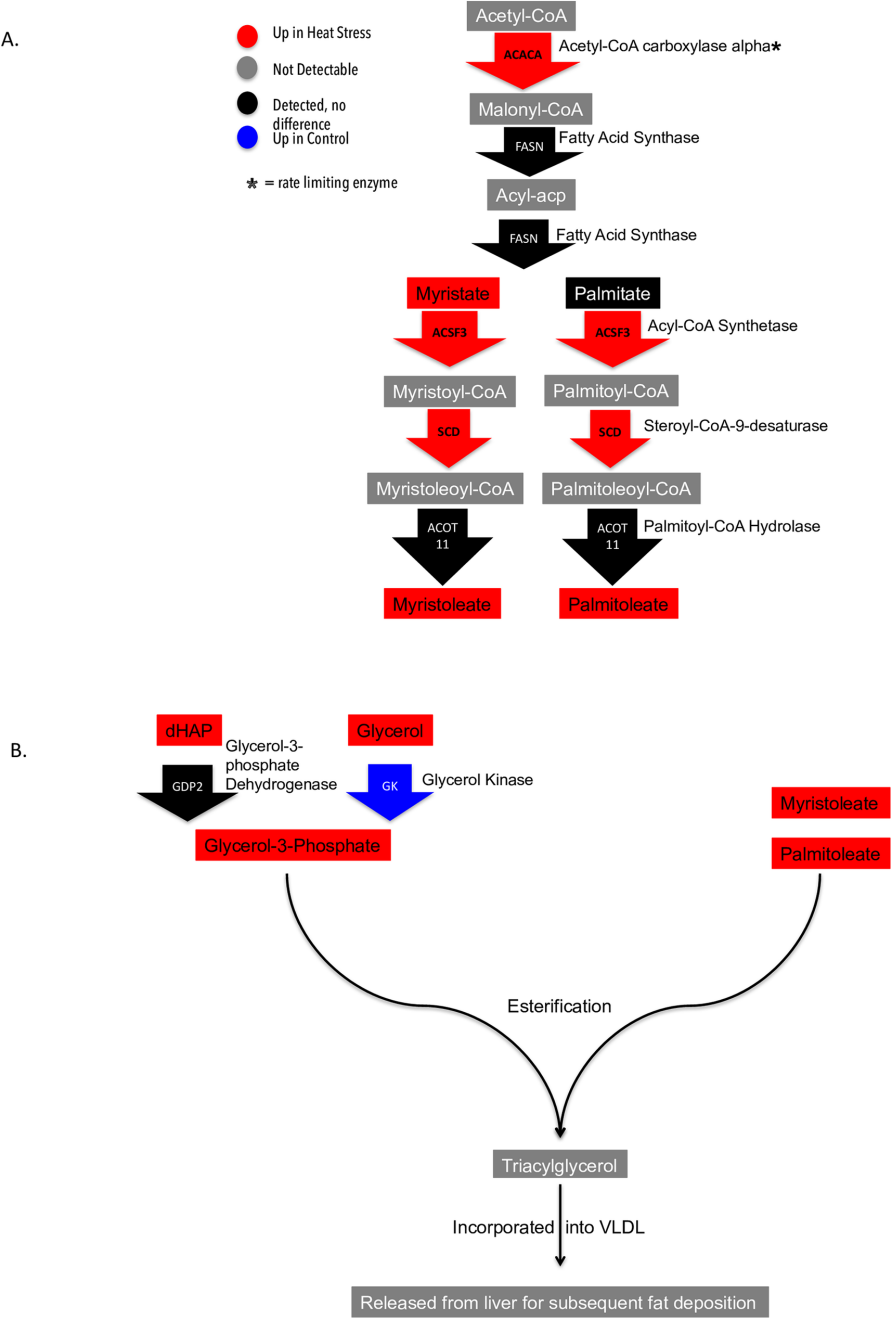
Depiction of fatty acid metabolism. **6a.** Integrated transcriptome and metabolome data and fatty acid metabolism depicting acetyl-coa being transformed into either myristate or palmitate. **6b** depicts the numerous ways glycerol-3-phosphate can be synthesized as well as the final esterification step to form a complete triacylglycerol.

Fatty acids serve many important metabolic functions. In the body, they are mostly stored as triglycerides, where they can be broken down for various uses. The heart also uses triglycerides as an energy source. Both transcriptome and metabolome data support increased fatty acid synthesis during heat stress. Table 1 lists the long chain fatty acids identified in the data and whether they are enriched or down-regulated during heat stressor unchanged. Most of the fatty acid levels were either down-regulated or unchanged, except for myristate, myristoleate, and palmitoleate, which were elevated. A number of genes encoding for enzymes at various phases of fatty acid synthesis were also elevated in heat stress (Fig 6). Acetyl-CoA carboxylase alpha (ACACA), which converts acetyl-CoA to malonyl-CoA was elevated in heat stress. Fatty acid synthase (FASN), which converts malonyl-CoA to acyl-ACP and then converts acyl-ACP to myristate or palmitate, was detected but unchanged between conditions. Acyl-CoA synthetase (ACSF3), which converts myristate to myristoyl-CoA and palmitate to palmitoyl-CoA was elevated in heat stress. Stearoyl-CoA-9-desaturase (SCD), which converts myristoyl-CoA and palmitoyl-CoA to myristoleoyl-CoA and palmitoleoyl-CoA respectively, was also elevated in heat stress. Palmitoyl-CoA hydrolase (ACOT11), which converts the latter metabolites to myristoleate or palmitoleate, was detected but unchanged. Glycerol and glycerol-3-phosphate (G3P) levels were also elevated in heat stress, the latter making up the triacylglycerol backbone. However, glycerol kinase (GK) levels were decreased during heat stress. An alternative hypothesis is that G3P is being synthesized through glyceroneogenesis. This is the conversion of precursors other than glycerol or glucose to G3P such as pyruvate, which is enriched in the heat stress sample set. There is evidence in mammals that glyceroneogenesis happens more often in the liver than glycerol conversion [13]. The last step in this process involves synthesizing G3P from dihydroxyacetone phosphate (dHAP). Although not achieving significance, dHAP is reduced under heat stress. The enzyme that performs the conversion from dHAP to G3P is glycerol-3-phosphate dehydrogenase (GDP2) and was detected but unchanged between conditions. A reasonable hypothesis is that equilibrium levels of substrates drives the reaction towards synthesizing G3P, leading to lower levels of available dHAP in birds. Once G3P and either myristoleate or palmitoleate undergo esterification, they are incorporated into very low-density lipoproteins (VLDL), shunted out of the liver for fat deposition, and transformed into low-density lipoproteins (LDL). The transcriptome data indicates increased levels of low density lipoprotein receptor class A domain containing 3 (LDLRAD3) gene, which encodes for a LDL receptor and decreased levels of very low density lipoprotein receptor (VLDLR), which encodes for a VLDL receptor, which is consistent with increased VLDL production and endocytosis of exogenous LDL.

**Table 1:** Fatty acids from Ross708 liver samples 28 days post-hatch and whether they are enriched in thermoneutral, heat stress, or unchanged between conditions.

Fatty Acid Metabolite	Enriched Condition
Arachidate	Thermoneutral
Erucate	Thermoneutral
Margarate	Thermoneutral
Nonadecanoate	Thermoneutral
Pentadecanoate	Thermoneutral
Myristate	Heat stress
Myristoleate	Heat stress
Palmitoleate	Heat stress
10-hedeptadecenoate	Unchanged
10-nonadecanoate	Unchanged
Eicosenoate	Unchanged
Palmitate	Unchanged
Stearate	Unchanged

The fatty acids enriched in thermoneutral are largely dietary derived fatty acids. Fatty acids enriched in heat stress are important in the production of triacylglycerols.

#### Glutathione metabolism and pentose phosphate pathway

**Fig 7:**
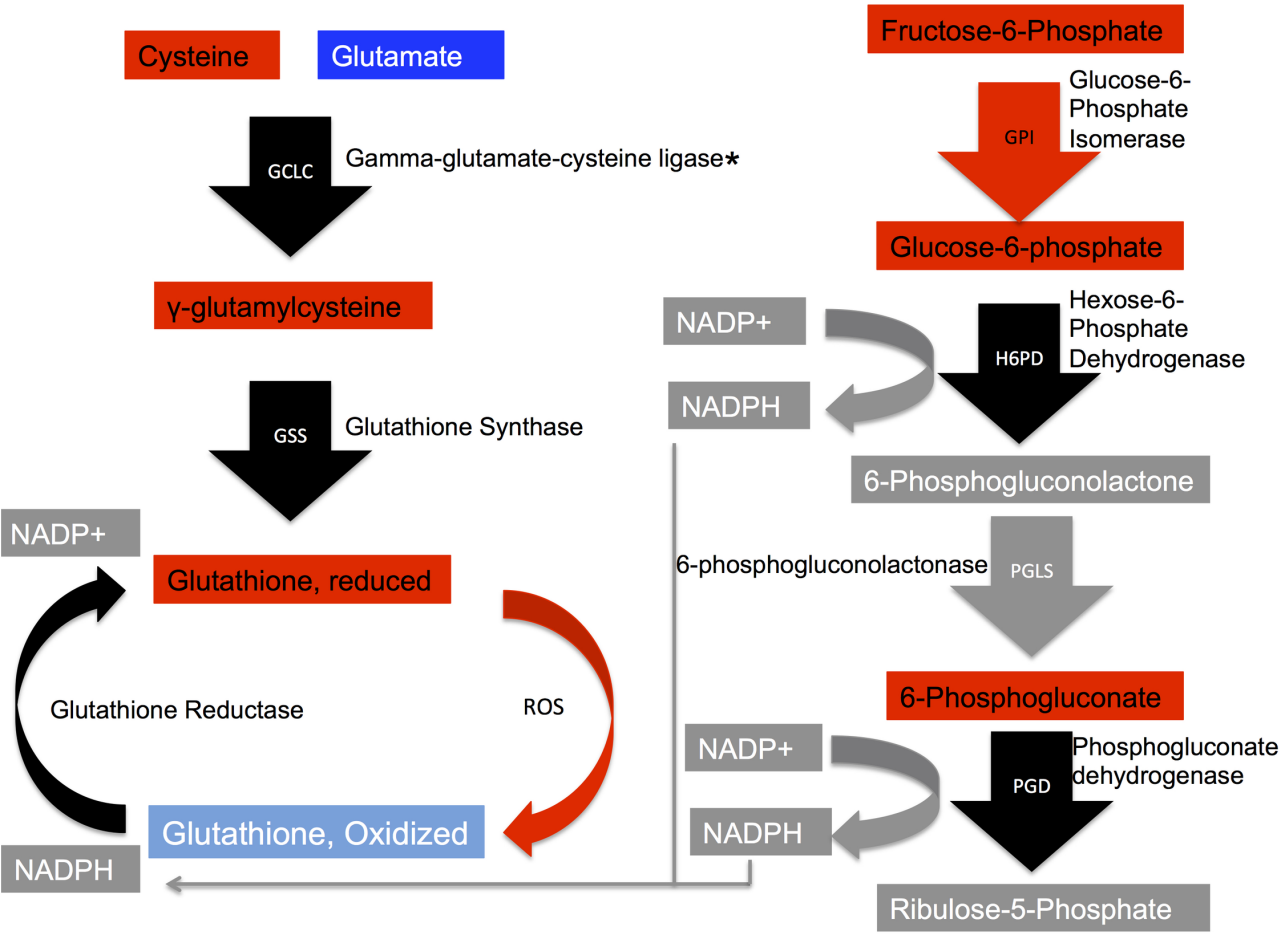
Left side of diagram is a depiction of glutathione metabolism. The right side of the diagram is a depiction of the pentose phosphate pathway, which generates the NADPH needed to transform oxidized glutathione back to the reduced form.

The main source of energy for the liver is amino acids [14]. Table 2 lists the detected free amino acids and whether they were elevated or reduced in heat stress, or unchanged. Most were reduced under heat stress, except cysteine, which was elevated in heat stress. Due to increased metabolic demands during heat stress, the liver likely increases breakdown of amino acids for energy. Cysteine is involved in glutathione metabolism, which is integral in preventing damage to cells caused by reactive oxygen species, a common sequela of heat stress[4, 15]. The concentration of available cysteine regulates the rate at which reduced glutathione is synthesized [16–18]. An increased level of cysteine is consistent with the observed increase in reduced glutathione under heat stress. The liver is a major source of circulating glutathione [19] and birds with elevated circulating levels of glutathione exhibit less oxidative damage under heat stress compared to birds with normal circulating levels [5]. The metabolome data also indicated an increased ratio of reduced glutathione (GSH) to oxidized glutathione (GSSG) in heat stress (3.56) compared to thermoneutral (1.19). This increased ratio is due to a marked increase in GSH levels, as the GSSG levels stayed fairly constant. Studies investigating acute heat stress indicate a decrease in GSH/GSSG ratios due to increased oxidative damage [20][21]. This study did not assess the impact of chronic heat stress on glutathione ratios. After a week of cyclic heat exposure, perhaps the liver has adapted to maintaining elevated levels of GSH to compensate for periodic heat stress. Fig 7 depicts glutathione metabolism as well the pentose phosphate pathway. Cysteine, and glutamate, which is reduced under heat stress, are transformed to gamma-glutamylcysteine (elevated in heat stress) by gamma-glutamate-cysteine ligase (unchanged). Glutathione synthase, which transforms gamma-glutamylcysteine to GSH, remains unchanged during heat challenge. Members of the pentose phosphate pathway (PPP) are also elevated in heat stress as well, including F6P and G6P. The PPP creates NADPH, which is used to convert the oxidized form of glutathione to the reduced form. Glucose-6-phosphate isomerase (GPI), which transforms F6P to G6P in the PPP is also elevated in heat stress. The enzyme that converts G6P to 6-phosphogluconolactone, hexose-6-phosphate dehydrogenase, is detected but unchanged. While 6-phosphogluconolactone is not detected, 6-phosphogluconate is elevated in heat stress. 6-phosphogluconolactonase has not yet been identified in the Gallus genome. Ribulose-5-phosphate is undetected but phosphogluconate dehydrogenase is detected and unchanged [22]. Possibly, elevated pentose phosphate pathway activity yields sufficient NADPH to convert oxidized glutathione to reduced glutathione thereby meeting the need to alleviate oxidative stress.

**Table 2:** Free amino acids detected by Metabolon and whether they are enriched in thermoneutral, heat stress, or unchanged between conditions from Ross708 liver samples 28 days post-hatch.

Amino Acid	Enriched Condition
Alanine	Thermoneutral
Arginine	Thermoneutral
Aspartate	Thermoneutral
Glutamate	Thermoneutral
Glutamine	Thermoneutral
Glycine	Thermoneutral
Histidine	Thermoneutral
Isoleucine	Thermoneutral
Lysine	Thermoneutral
Methionine	Thermoneutral
Phenylalanine	Thermoneutral
Proline	Thermoneutral
Serine	Thermoneutral
Threonine	Thermoneutral
Tryptophan	Thermoneutral
Valine	Thermoneutral
Cysteine	Heat stress
Asparagine	Unchanged
Leucine	Unchanged*
Tyrosine	Unchanged*

All amino acids are enriched in thermoneutral or approaching significantly enriched in thermoneutral except asparagine, which is unchanged, and cysteine, which is enriched in heat stress.

*Indicates approaching significantly enriched in thermoneutral

#### Beta-oxidation

Beta-oxidation is an energy and heat producing pathway. It breaks down fatty acids in the mitochondria to produce acetyl-CoA for the citric acid cycle and NADH and FADH2 for the electron transport chain. The metabolomic data indicate increased levels of Coenzyme A under heat stress (S1 Fig), which is covalently attached to fatty acids for transport into the mitochondria. However, there are decreased levels of the acyl carnitines involved in transport of fatty acids in the beta-oxidation pathway under heat stress. The elevated levels of uncharged coenzyme A and reduced levels of acyl carnitines suggest that fewer substrates are being delivered to the mitochondria for beta-oxidation. This would result in reduced body temperature and shifting the balance between lipolysis and lipogenesis in favor of lipogenesis.

#### Glycosylation

One metabolite that was highly enriched in heat stress was mannose-6-phosphate (S2 Fig). This metabolite is involved in a number of different biological processes, including glycosylation. The genes that catalyze conversion of mannose-6-phosphate to mannose-1-phosphate, phosphomannomutase 1 and phosphomannomutase 2 are both enriched in heat stress. This process is important for the production of GDP-mannose, which is essential for N-linked glycosylation. There is evidence for increased glycosylation under acute heat stress, likely to regulate heat shock proteins. One study indicated calreticulin as being one of the first major glycosylation products of heat stress [23]. It is detected in our data but unchanged between conditions. The function of glycosylation under heat stress is still largely unknown [24].

#### Endocannabinoids

Endocannabinoids were reduced in heat stress samples. While not well studied in birds, in mammals the endocannabinoids function in appetite regulation, lipogenesis, metabolism, and signaling. While their primary function is in the brain, they also function in peripheral tissues [25]. There is decreased weight gain and lipogenesis when the cannabinoid receptor is knocked out in mice livers [26] while injected cannabinoid receptor agonists, causes weight gain and increased lipogenesis in mice [26]. Perhaps heat stress reduces endocannabinoids in the liver, thereby reducing the level communicated to the brain. This could be an important cause for the reduction in feed consumption seen under heat stress. Contrary to the effects seen in mouse mutants, the chicken liver appears to shift towards increase lipogenesis under heat stress. Possibly, the multiple stimuli produced by heat stress overrides the tendency of reduced endocannabinoid levels to increase decrease lipogenesis. More research is needed to identify the impact of reduced endocannabinoids under heat stress.

## Conclusions

Overall, the liver appears to produce a robust response to heat stress after one week of cyclic heat stress, maintaining homeostasis and preventing damage due to oxidative stress. Transcriptome and metabolome together provide convincing evidence for increased glycogenolysis and gluconeogenesis as well as fat deposition, glycosylation, and glutathione production under heat stress, (S3 Table). There also appears to be inhibition of cell cycle perhaps to allow time for DNA damage caused by heat stress to be repaired. The inflammatory system appears to be suppressed in heat stress along with lipolysis, beta-oxidation, and endocannabinoid synthesis. There are seven genes involved in circadian rhythm affected by heat stress. Syncing circadian response to heat stress could be a fundamental adaptation to a cyclic heat stress response.

## Materials and methods

### Ethics statement

This study was carried out in strict accordance with the recommendations in the Guide for the Care and Use of Laboratory Animals of the National Institutes of Health. The protocol was approved by the Committee on the Ethics of Animal Experiments of the University of Delaware (Permit Number: 2703-12-10).

### Bird and tissue handling

Male broiler chickens *(Gallus gallus)* were obtained from Mountaire hatchery (Millsboro, DE) on day of hatch and divided into thermoneutral and experimental houses on the University of Delaware farm. They were raised under a light cycle of 23 hours of light and 1 hour of dark. Standard management and husbandry procedures were followed, as approved by the Animal Care and Use Committee (AACUC #(27) 03-12-14R). Birds were given *ad libitum* access to water and fed the same diet (corn-soy) which met all NRC requirements [27]. Both groups were raised at 35°C until one-week post hatch. Temperature was decreased 5°C each week thereafter until temperature reached 25°C at day 21 post hatch. The thermoneutral house was then maintained at 25°C and the heat stress house was subject to 35–37°C for 8 hours per day, to mimic an environmental heat wave. Temperature in both houses was maintained by a computerized system controlling heaters and ventilation fans (Chore-time Equipment, Milford, Indiana). Temperature ranged between 35–37°C during the eight hours of heat stress. This yields an internal body temperature (cloacal) of 43.5°C within two hours of the onset of heat stress. This body temperature can induce a heat stress response in chicken cells [6]. In the control (thermoneutral) house the temperature ranged between 23–25°C during this same period. Both houses were maintained at 23–25°C during the thermoneutral period (16 hours) of the day. Birds were euthanized via cervical dislocation and necropsied at day 28 post hatch, following one week of cyclic heat stress. Livers were flash frozen in liquid nitrogen, and stored at -80°C for further processing.

### RNA and library preparation

Forty-five mg of the left lobe of 8 thermoneutral and 8 heat stress liver samples were homogenized and RNA was extracted using the mirVana miRNA Isolation Kit (Ambion, Austin, TX) as per manufacturer instructions. They were quantified using the Qubit 2.0 Fluorometer (Qubit, New York, NY). Samples were checked for quality using the Fragment Analyzer (Advanced Analytical, Ankeny, IA) at the Delaware Biotechnology Institute (DBI, Newark, DE). Libraries were made using the Illumina TruSeq Stranded mRNA Sample Preparation Kit (Illumina, San Diego, CA) per manufacturer instructions and sent to DBI for sequencing.

### Metabolome sample preparation

Fifty mg of 12 thermoneutral and 11 heat stress liver samples were sent to Metabolon (Durham, NC), for analysis of the metabolome. All of the samples used for the transcriptome analysis were included in the metabolomic sample set. Samples were analyzed as previously described [28]. Samples were prepared using the MicroLab STAR system from Hamilton Company (Reno, NV) using in house recovery standards prior to extraction for QC purposes. Extract was divided into fractions for two reverse phase (RP)/UPLC-MS/MS methods (positive and negative ion mode electrospray ionization), and one for HILIC/UPLC-MS/MS with negative ion mode ESI. Several control were used, including the use of technical replicates, extracted water samples as blanks, and in house QC samples to monitor chromatographic alignment. All UPLC-MS/MS methods used a waters ACQUITY UPLC and Thermo Scientific Q-Exactive high-resolution mass spectrometer. Each sample extract was dried and reconstituted with solvents compatible to each method and solvents included a series of standards at fixed concentrations. Metabolon used hardware and software extract created by the company to extract, peak-identify, and QC process the raw data. Compounds were identified using a Metabolon maintained library of purified standards or recurrent unknown entries. Data is provided as a supplementary.txt file (S1 Data). Over 3300 compounds have been identified and registered in Metabolon’s library. The data was statistically analyzed using a Welch’s two-sample t-test following a log transformation and imputation of missing values with the minimum observed value for each compound. The company provided an analysis that included pathway visualizations. These pathway analyses were then incorporated with the transcriptome data to create a more complete view of changing pathways.

### Transcriptome analysis

Once libraries were sequenced, data were processed using an in-house pipeline and fragments per kilobase per million mapped reads (FPKM) values were determined. The sequencing data is publicly available through GEO series accession number GSE95088 (https://www.ncbi.nlm.nih.gov/geo/query/acc.cgi?acc=GSE95088).

Differentially expressed genes were determined by first taking the mean FPKM value for each condition, then excluding any genes where both the heat stress and thermoneutral means were less than 1. Then, the log2 ratios of the mean of heat stress divided by thermoneutral were taken, and subjected to a t-test to determine significance. Only genes with a p<0.05 were considered for subsequent analyses. Genes were then input to AmiGO2 [29], for gene ontology (GO) terms. We then used Pathrings [30], for pathway analysis, and WebGIVI [31], a text-mining tool that relates gene lists to literature through the use of iTerms.

## Supporting information

S1 Table AmiGO2 ontology results for the enriched genes from 28-day-old broiler chicken livers after one week of cyclic heat stress sorted by p-value. This list only includes significant ontological terms.
(XLSX)

S2 Table AmiGO2 ontology results for enriched genes from 28-day-old broiler chicken livers in the thermoneutral condition enriched genes sorted by p-value. This list only includes significant ontological terms.
(XLSX)

S3 Table List of genes integrated with metabolomic data sorted by pathway. Included in this list are the gene symbol, gene name, fold change, p-value, and pathway affected. This list includes genes that are significantly different between heat stress and thermoneutral conditions, not significantly different between conditions, and genes not detected in the genome.
(XLSX)

S1 Fig Beta-Oxidation pathway with metabolomic data overlay from 28-day-old broiler chicken livers after one week of cyclic heat stress, provided by Metabolon. Circles indicate metabolites and squares indicate enzymes. Red indicates enrichment in heat stress condition and blue indicates enrichment in the thermoneutral condition. Black indicates metabolite is detected but unchanged between conditions and Gray indicates not detectable. Reprinted from Metabolon under a CC BY license, with permission from Metabolon, original copyright 2015.
(TIF)

S2 Fig Glycosylation pathway with metabolomic data overlay from 28-day-old broiler chicken livers after one week of cyclic heat stress, provided by Metabolon. Circles indicate metabolites and squares indicate enzymes. Red indicates enrichment in heat stress condition. Black indicates detected but unchanged between conditions. Grey indicates not detectable. Reprinted from Metabolon under a CC BY license, with permission from Metabolon, original copyright 2015.
(TIF)

S1 Data Metabolic data provided by Metabolon Inc. (TXT)
