# Supplementary figures and images for: Chicken hepatic response to chronic heat stress using integrated transcriptome and metabolome analysis
Source: PLoS One. 2017 Jul 31;12(7):e0181900. doi: 10.1371/journal.pone.0181900 (PMC5536301; doi:10.1371/journal.pone.0181900)

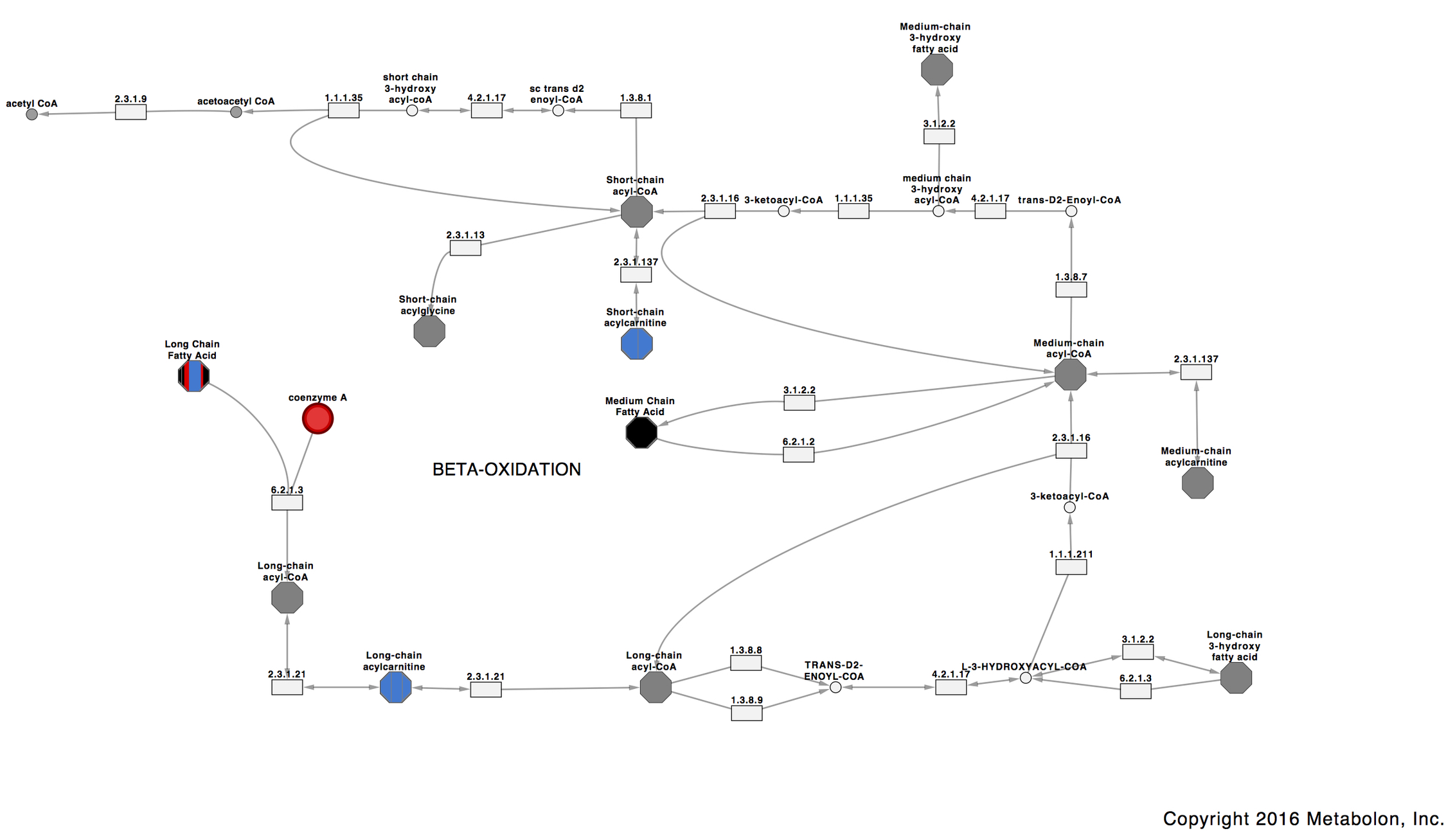

Supplement: S1 Fig — Circles indicate metabolites and squares indicate enzymes. Red indicates enrichment in heat stress condition and blue indicates enrichment in the thermoneutral condition. Black indicates metabolite is detected but unchanged between conditions and Gray indicates not detectable. Reprinted from Metabolon under a CC BY license, with permission from Metabolon, original copyright 2015. (TIF) [file pone.0181900.s004.tif]

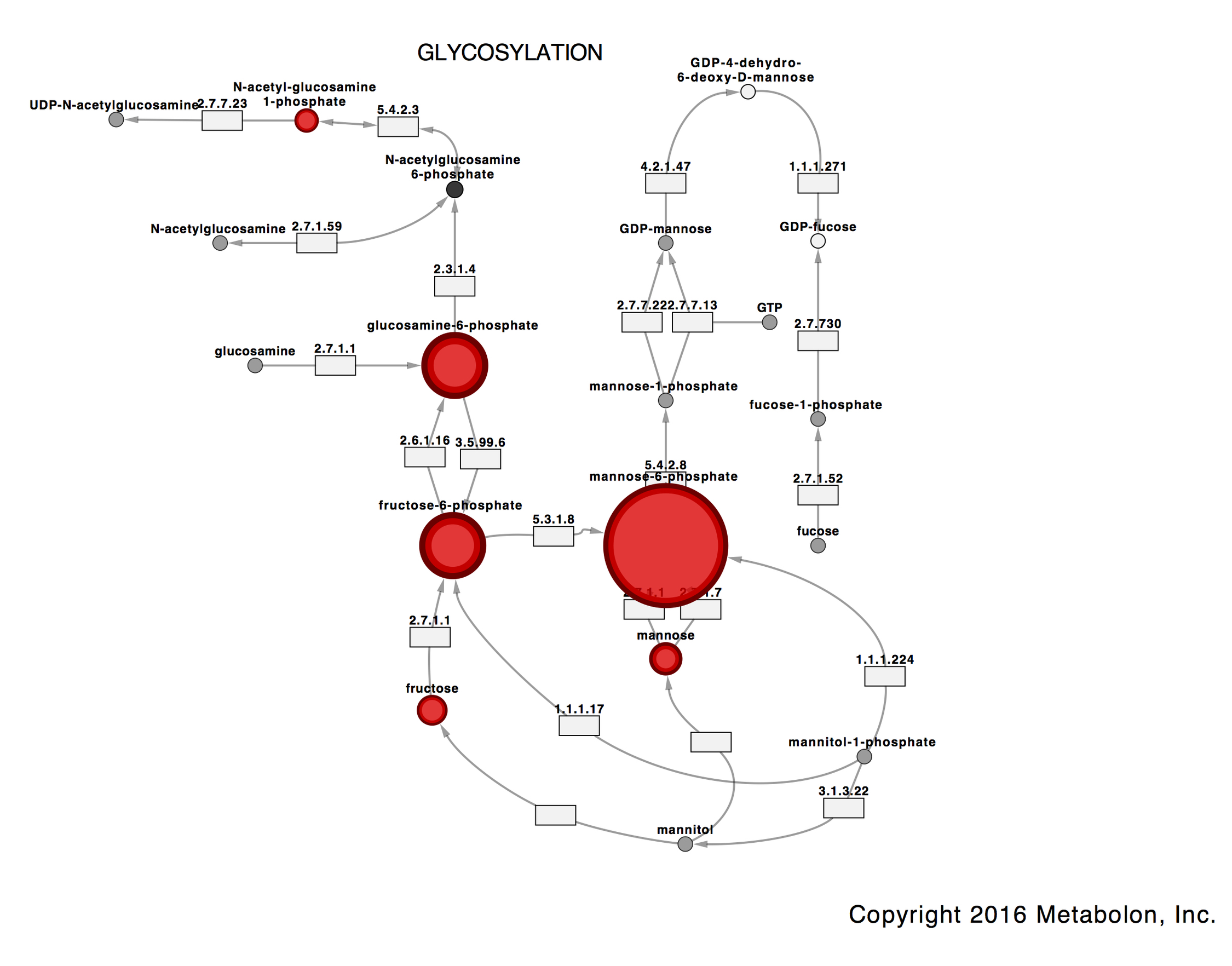

Supplement: S2 Fig — Circles indicate metabolites and squares indicate enzymes. Red indicates enrichment in heat stress condition. Black indicates detected but unchanged between conditions. Grey indicates not detectable. Reprinted from Metabolon under a CC BY license, with permission from Metabolon, original copyright 2015. (TIF) [file pone.0181900.s005.tif]
